# Modeling the competition between lung metastases and the immune system using agents

**DOI:** 10.1186/1471-2105-11-S7-S13

**Published:** 2010-10-15

**Authors:** Marzio Pennisi, Francesco Pappalardo, Ariannna Palladini, Giordano Nicoletti, Patrizia Nanni, Pier-Luigi Lollini, Santo Motta

**Affiliations:** 1 Department of Mathematics and Computer Science, University of Catania, V.le A. Doria 6, Catania, Italy; 2 Laboratory of Immunology and Biology of Metastasis, Cancer Research Section, Department of Experimental Pathology, University of Bologna, Bologna, Italy; 3 Laboratory of Experimental Oncology, Rizzoli Orthopedic Institute, Bologna, Italy; 4 Department of Hematology and Oncologic Sciences "L. e A. Seragnoli", University of Bologna, Bologna, Italy

## Abstract

**Background:**

The Triplex cell vaccine is a cancer cellular vaccine that can prevent almost completely the mammary tumor onset in HER-2/neu transgenic mice. In a translational perspective, the activity of the Triplex vaccine was also investigated against lung metastases showing that the vaccine is an effective treatment also for the cure of metastases. A future human application of the Triplex vaccine should take into account several aspects of biological behavior of the involved entities to improve the efficacy of therapeutic treatment and to try to predict, for example, the outcomes of longer experiments in order to move faster towards clinical phase I trials. To help to address this problem, MetastaSim, a hybrid Agent Based - ODE model for the simulation of the vaccine-elicited immune system response against lung metastases in mice is presented. The model is used as in silico wet-lab. As a first application MetastaSim is used to find protocols capable of maximizing the total number of prevented metastases, minimizing the number of vaccine administrations.

**Results:**

The model shows that it is possible to obtain "in silico" a 45% reduction in the number of vaccinations. The analysis of the results further suggests that any optimal protocol for preventing lung metastases formation should be composed by an initial massive vaccine dosage followed by few vaccine recalls.

**Conclusions:**

Such a reduction may represent an important result from the point of view of translational medicine to humans, since a downsizing of the number of vaccinations is usually advisable in order to minimize undesirable effects. The suggested vaccination strategy also represents a notable outcome. Even if this strategy is commonly used for many infectious diseases such as tetanus and hepatitis-B, it can be in fact considered as a relevant result in the field of cancer-vaccines immunotherapy. These results can be then used and verified in future "in vivo" experiments, and their outcome can be used to further improve and refine the model.

## Background

The metastatic process is an extraordinary complex process. In order to colonize a secondary site and to become metastases cancer cells must complete a sequential chain of steps which include the detachment from the primary tumor, the invasion through surrounding tissues and basement membranes, the survival in the circulation, lymphatics or peritoneal space and the settlement in a distant target organ. In spite of this intrinsic inefficiency, metastases represent one of the major concerns in the clinical management of cancer. The majority of cancer mortality is associated with this disseminated disease rather than the primary tumor [1]. In most cases cancer patients with localized primary tumors have significantly better prognoses than those with disseminated tumors. Recent evidence shows that metastases can be an early event [2] and that 60% to 70% of patients have already initiated the metastatic process by the time of diagnosis. Even patients that have no evidence of tumor dissemination at diagnosis are at risk from metastatic disease. Approximately one-third of women who are sentinel lymph node negative at the time of surgical resection of the primary breast tumor will develop clinically detectable secondary tumors [3].

Transgenic mice are preclinical models which develop autochthonous tumors and are interesting for cancer preventive studies since they reproduce the natural evolution of tumors. HER-2/neu transgenic mice are likely the most extensively studied models for the evaluation of approaches against mammary carcinomas that spontaneously develop over the course of several months. One of these mice systems is represented by BALB-neuT female mice, which start to develop after birth cells hyper-expressing HER-2/neu gene product (p185) in mammary glands. These cells give rise to multiple microscopic lesions identifiable as atypical hyperplasia, which progress to carcinomas in situ, becoming macroscopic lesions detectable at around 4-5 months of age.

One of the most effective vaccines to prevent the onset of mammary tumors was set up in BALB-neuT female mice. This vaccine, referred to as Triplex [4,5], was obtained from a mammary carcinoma of a FVBneuN #202 (H-2q) mouse, transgenic for the rat protooncogene c-neu, and combines three different stimuli:

• The p185neu antigen, product of the rat HER-2/neu gene;

• H-2q MHC molecules (allogeneic for H-2 d BALBneuT mice);

• Interleukin-12 (the cells are engineered with the genes coding for murine IL-12).

The Triplex vaccine demonstrated an ability to prevent almost completely neu-driven mammary carcinogenesis [4,5]. In order to predict the best vaccination protocol a computational model, hereafter referred as SimTriplex model [6], has been successfully used in conjunction with Genetic Algorithms [7]. In a translational perspective, the therapeutic activity of the Triplex vaccine was evaluated in BALB-neuT mice against different stages of mammary carcinoma progression. The study of therapeutic activity of the Triplex vaccine showed that the vaccine loses progressively its efficacy with the advancement of tumor progression in BALB-neuT mice with little or no efficacy at all against incipient mammary carcinomas. On the other hand the Triplex vaccine proved to be an effective treatment against induced lung metastases [8]. Lung metastases were induced in BALB-neuT mice by intravenous injection of syngeneic mammary carcinoma cells.

The administration of the vaccine started one day after the intravenous injection of the metastatic cells and it is repeated twice weekly up to the end of the experiment (day 32), with lower but good prevention rates when the same cycle is started 7 days after the induction of the metastases (Triplex+7 protocol). The immunological responses in the immunoprevention and therapeutic experiments overlap only partially. A major goal of biologists is to better understand the biological behavior to improve the efficacy of the therapeutic treatment and to try to predict, for example, the outcomes of longer experiments in order to move faster towards clinical phase I trials. Following previous experiences [9,10] we then developed a new computational model named MetastaSim to be used as an *in silico *virtual lab can help answering these questions. The model was briey introduced and presented as work-in-progress in [11].

The MetastaSim model can be seen as an agent-based (ABM) or automata-like model, and it has been inspired by the SimTriplex model. MetastaSim has in common with SimTriplex the same modeling framework and some of the biological mechanisms shared by the in vivo experiments they model. However it has some important differences.

The first differences are determined by the biological, spatial and time-length differences entitled with the two in vivo experiments. The immunoprevention experiment lasts for 1 year whereas the therapeutic experiment lasts for 1 month. SimTriplex simulates only a small fraction of a mammary gland (1 *mm*^3^) whereas MetastaSim reproduces the frontal ventral surface of the left lung of a mouse, for an estimated volume which is 64 times bigger than those simulated by SimTriplex. The biological behavior is also different. Cancer prevention is primarily driven by antibodies. Interferon-γ (IFN-γ) is the one of the major mediators both in cancer prevention and in the metastasis therapy whereas anti-HER-2/neu antibodies, which are key effectors of the Triplex preventive ability, seem devoid of significant therapeutic activity. Other vaccine-induced mechanisms playing a causal role in the therapeutic experiment are represented by T-helper activities at the systemic level and macrophages infiltration in the tumor cell nests.

Apart from the aforecited physical and biological differences, MetastaSim introduces some important advances in respect to its predecessor. The most important improvement the model presents consists in a complete revision of the cancer growth kinetics. The model is now able to simulate multiple different metastatic nodules, each one with its own growth rate, more accurately. To reproduce the growth in time of nodules, the Gompertz growth law is now used in its differential form, making the model an ABM - ODE hybrid one. The growth rates are randomly generated in such a way that the in silico nodule measurements resemble the experimental measurements coming from the in vivo experiment. Moreover a first simplified chemotaxis and some of the effects of the immunosuppression induced by cancer cells have been introduced into the model as well, in order to make the model more consistent with the last immunological advances. After a tuning phase, necessary to determinate the free parameters, the model has been validated against existing in vivo experiments. Then its first application has been to find a protocol capable to assure against lung metastases the same protection entitled with the use of the Triplex+1 protocol.

One of the major concern of the biologists is in fact to understand wether it is possible to gain a similar protection of the Triplex+1 protocol with less injections. Wet biology requirements (i.e., the time required for the preparation of the vaccine) as well as safeness for the mice (i.e., the need to avoid undesirable effects) entitle that no more than two vaccinations per week (in pre-established days) can be done. This means that for the length of the "in vivo" experiment (32 days) it is possible to administer the vaccine only in 9 days. It turns out that the Triplex+1 protocol, which already counts 9 injections, is the most intensive one because it already uses all the available days to vaccinate. Shorter protocols should be therefore obtained by removing some injections from Triplex+1 protocol.

## Methods

### The in vivo experiment

In the immunoprevention experiment, BALB-neuT mice that have reached 6 weeks of age are exposed to a twice weekly intraperitoneal vaccination cycle followed by two weeks of rest (**Chronic **protocol) for one year. At the end of the experiment the number of detectable lesions is taken as the final outcome. In the therapeutic experiment, instead of waiting for breast cancer to develop into its later stages (that give rise to lung metastases), metastases are induced by the injection of metastatic cells. Mice in late tumor stages present various problems, such as immune system aging, the presence of a non surgically removable primary tumor and the inability of establishing when the metastatic process starts. Indeed the experimental induction of metastases in tumor-free mice clearly represents a typical scenario in human cancer, i.e., the scenario arising after the surgical removal of the primary tumor.

The therapeutic experiment lasts for 32 days. TuBo mammary carcinoma neu cells (referred to as Neu/H-2) are used to induce experimental metastases in syngeneic BALBneuT mice. At day 0 all mice receive an intravenous injection of 2.5·10^4 ^metastatic TuBo cells.

This experiment counts three different mice sets: the untreated or control set, "set I", where a protocol composed by a "twice a week" vaccination cycle is started at day 1 and repeated up to the end of the experiment (Triplex+1 protocol), and "set II", where the same cycle is started at day 7 and repeated up to the end of the experiment (Triplex+7 protocol).

Mice from the control set developed ≈ 200 metastatic nodules, whereas mice from set I and II shown a reduction > 99% and ≈ 87% of early lung metastasis formation respectively. Moreover, the structure of metastatic lesions was frequently cribriform and less compact than in the controls.

The three stimuli of the triplex vaccine stimulate the immune system responses in many ways. The IL-12 enhances antigen presentation, helper T cell (TH) activation and secretion of interferon-γ (IFN-γ) by natural killer (NK) and TH cells. IFN-γ also has a cytostatic activity on cancer cells (CC) and stimulates granulocytes and macrophages (MP) in infiltrating tumor cell nests in the lungs. TH cells have a major role at the systemic level releasing various cytokines such as interleukin-2 (IL-2) which enhances cytotoxic T cells (TC) activities and B cells antibodies (Ab) release. The allogeneic MHC favors the recognition by antigen presenting cells (macrophages, B and dendritic cells (DC)), as well as cytotoxic T cells.

### General description of the MetastaSim computational model

The MetastaSim model uses an agent based approach to simulate the main features of the immune system. It takes inspiration from the SimTriplex model developed by Pappalardo et. al. [6]. Both the innate and adaptive immune responses are part of the model. In particular the core of the adaptive immune response is represented, and cellular and humoral responses as well as the presence of antigens in the host organism are implemented.

The space is discrete. MetastaSim uses an hexagonal lattice instead of the more familiar square (checkerboard) lattice. This is because the neighbors of a site in a square lattice are of two different types, edges and corners. To have only neighbors of one type we choose the hexagonal lattice where each site has six identical neighbors. The lattice represents the frontal ventral surface of the left lung of mice and it has periodic boundary conditions. The model does not include the presence of lymph nodes. However all the relevant entities and processes that occur in lymph nodes are represented.

Perelson and Oster (1979) [12] proposed a simple quantitative model to understand how large the immune repertoire should be. This model is based on the notion of *shape space*. In order for a receptor and the molecule that it binds, a ligand, to approach each other over an appreciable portion of their surfaces, there must be extensive regions of complementarity. The constellation of features important in determining binding among molecules is called the *generalized shape *of a molecule.

One of the key aspects of the model is represented by the use of bit-strings to model receptors. Bit-strings are excellent candidates to describe the concept of shape space. This fundamental modeling abstraction ignores nearly all of the physical details that determine receptor/ligand interactions. However, by adopting character strings, many binding events can be simulated quickly, making it feasible to study large-scale properties of the immune system [13].

Although character strings are unphysical, they can produce surprisingly accurate models when benchmarked to experiment [14], suggesting that the abstraction captures important features of receptor/ligand binding.

The time-step for the simulation is Δ*t *= 8 hours, since no other biologically relevant processes for the *in vivo *experiment can be observed in shorter times. At any time step the interaction and the diffusion processes hold.

During the interaction process the concept of physical proximity is modeled through the concept of lattice-site: only entities that lie in the same lattice-site can stochastically interact with each others in the same site, so that there is no correlation between entities residing on different sites at a fixed time.

After the interaction phase, entities can move from a lattice-site to another one in the neighborhood. Major bone marrow and thymus functions, like the positive and negative selection of immature T lymphocytes before they get into the lymphatic system, are also represented.

#### Cells and molecules

The model includes the major cell types needed to represent the immune responses elicited by the vaccine. The main involved types of cells and molecules represented are dendritic cells (DC), macrophages (M), natural killer cells (NK), vaccine cells (VC), cancer cells (CC), b lymphocytes (B), cytotoxic lymphocytes (TC), t-helper lymphocytes (TH), antibodies (Ab), antigens (Ag), immunocomplexes (IC), interleukin-2 (IL-2), interleukin-12 (IL-12) and interferon-γ (IFN-γ).

More than one entity can be present at the same time in a lattice site. Cells are followed individually throughout the course of an experimental run because their internal states and receptors follow their own, individual life histories.

All cells have MHC class I receptors. APCs (macrophages and dendritic cells) and B cells also have MHC class II receptors. Macrophages and dendritic cells have receptors (i.e., Toll-like receptors) used for recognizing antigens aspecifically. These aspecific receptors are not represented explicitly. B cells, TH cells and Tc cells are endowed with receptors used for binding specific antigens. The B cell receptor (BCR) binds antigen which it can then ingest and endocytose. The T cell receptors (TCR) only bind antigen in MHC/peptide complexes. When B cells become plasma cells, they have no specific receptor, however they produce antibodies with the same receptor shape as the B cell from which they are descended.

With respect to the internal states, it is possible to describe the cells as finite state machines. In particular, they can take a state from a certain set of suitable states and their dynamics are realized by means of state-changes. A state change takes place when a cell interacts with another cell or with a molecule. Molecules are represented in the model as site-specific concentrations, since they have only a fixed set of properties that does not change throughout the simulation. These molecules include antigens, antibodies and various cytokines.

Antigens can be schematized as molecular structures containing essentially two different portions: epitopes and peptides. Epitopes represent the external part of an antigen that is recognized by, for example, a B-cell receptor whereas peptides represent the internal portion of the antigen that can be bound by an MHC molecule, expressed on a cell surface, and recognized by the appropriate T cell.

Epitopes and peptides are represented in the model by two distinct arrays of binary strings. The minimum antigen is constituted by just two strings, one for the epitope and one for the peptide.

Antibodies are represented in the same way as antigens, i.e., bit-strings. Antibodies contain both foreign (arising from the variable regions) and self (arising from the constant regions) peptides. Lastly, they contain the Fc portion, an epitope identical for all antibodies that is not explicitly represented since it is the same for all of them.

The model also represents some important cytokines which are represented by enumerating their concentration (number of molecules) for each lattice-site.

#### Entities interactions

Every interaction is a complex process that usually involves entities state change. It is possible to distinguish between specific and aspecific interactions. Specific interactions require the presence of specialized receptors for the recognition phase, such as those of B and T lymphocytes. For these kinds of interactions the probability of recognition is defined as a function of the Hamming distance between the receptors, and the affinity of the interaction can be enhanced by adjuvants.

Aspecific interactions entail the use of aspecific receptors, so the recognition phase is not represented explicitly. When two entities that may interact, occur at the same lattice site, they interact probabilistically.

**Figure 1:**
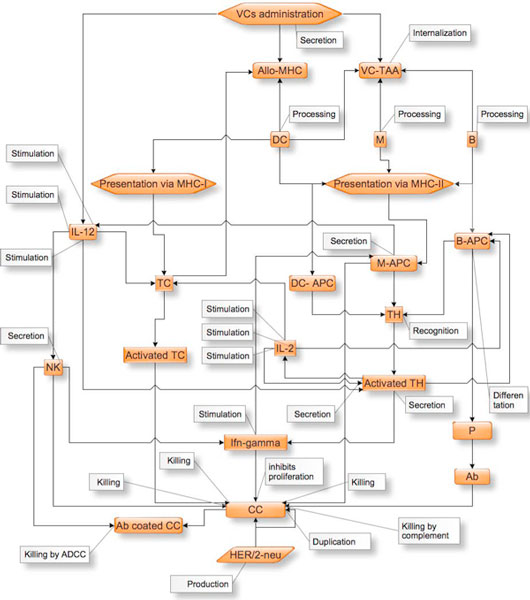
**The interactions network implemented in MetastaSim**. The generalized interactions scheme. Some minor entities and interactions are omitted to improve readability.

The network of interactions the model uses is represented in figure 1.

Vaccine cells (VC) can be directly recognized by TC cells and NK cells. The Allogenic MHC-I molecules favor the interaction with TC cells that proceed to kill them. NK cells can recognize VCs bounded by antibodies. Killed VCs release the tumor associated antigen (TAA) and IL-2.

The TAA can be captured by DC, MP and B cells that proceed to internalize the antigen and to present it with the MHC-II to TH cells. DC cells can also cross-present the antigen in conjunction with MHC-I to TC cells.

TH cells are activated by the interaction and proceed to release IL-2. B cells are also stimulated by interaction with TH cells to differentiate into Plasma cells (P) or, eventually, into Memory B cells. P cells release antibodies (Ab) which can bind both Vaccine and Cancer cells favoring NK killing activity. NK cells can also kill CCs that under-express the MHC complex.

Abs can also bind the antigen giving rise to Immunocomplexes (IC) that are then phagocyted by MPs. In addition they can activate the complement system with lysis of CCs as result. IL-2 promotes TC and TH activities, and B differentiation.

IL-12 promotes NK activities and TC activation. Moreover it stimulates TH cells and (to a lesser extent) NK cells to release IFN-γ. IFN-γ has cytostatic effects on CCs and stimulates both the killing of CCs and the release of IL-12 by MPs.

It is easily understandable that the complex network of interactions presented here has many cycles in it, i.e., TH ↔ IL-2. These cycles usually involve non-linearities that the model is however able to handle without any problem.

#### How simulation proceeds

The first step of the simulation consists of initialization. After cells generation and thymic selection processes, the grid is populated by randomly placing the various cell types in the lattice.

Interactions take place between entities that occupy the same site, and all entities have the opportunity to interact at every time-step. Ideally all the interactions should be executed at the same time. This is obviously not possible. Since the interactions dynamics of a lattice-site is not influenced by those of the other sites, the only problem is to decide how to manage interactions that occur at the same site.

At any time-step and for every lattice-site an interaction scheme is generated by choosing a random order for the interaction rules, with the interactions executed as defined by interaction scheme. Given a specific interaction *A *↔ *B *between entities of type *A *and *B*, every entity of type *A *is compared with all the entities of type *B *in the same site until a successful interaction occurs. Then, the next entity of type *A *is taken into consideration and it is compared again with all the entities of type *B *in the same lattice site. When all the entities have had their chance to interact, the next interaction rule in the interaction scheme is examined.

It is here that the concentration dependence of the model arises. For example, an APC may only have an antigen binding strength of 0.001, but if there are 100 antigens in the site the APC will have an opportunity to bind each of them. The resulting probability that the APC will actually bind an antigen will be approximately 0.1.

The next step after the interaction phase is represented by entity propagation. All the entities have a chance to move from the lattice site where they are to another one in the neighborhood. All the lattice sites have normally the same probability of being chosen as new positions. However, in a first attempt to mimic chemotaxis, higher probabilities are given to sites containing a congruous number of cancer cells. This "simplified-chemotaxis" is only active for some entities such as, for example, TCs and MPs.

Other minor processes related to non interaction-driven dynamics such as aging and natural death, differentiation and mutation, are then executed. Time step interactions will proceed up to a preselected final time.

#### Modeling of the metastatic growth pattern using the Gompertz growth law

The untreated mice scenario represents the development of the metastatic burden in untreated mice. It is the most computationally expensive scenario since, due to the lack of vaccination, the number of cancer cells grow enormously (≈ 10^7 ^different cells in each simulation) resulting in a memory intensive application. For modeling the development of the metastatic burden two strictly connected problems arose:

• How to obtain in the model nodule sizes whose distribution is somewhat similar to the *in vivo *experiment.

• How to represent the growth pattern of the nodules during the simulation.

In the *in vivo *experiment, keeping track of the temporal evolution of nodules sizes is not achievable, because to measure the nodules mice have to be killed and they cannot obviously continue in the experiment. Thus, to represent the temporal growth kinetics of nodules, some assumptions were made. Biologists firstly assumed that every metastatic nodule has originated from an individual "progenitor" cancer cell. This means that approximately only one cancer cell in 100 passes through lung capillary vessels and settles into the lungs showing, indeed, how inefficient the metastatic process is.

We note here that, since the positioning process is not relevant for the experiment, the simulation starts with cancer cells already settled in the lungs. At the beginning of the simulation *n *"progenitor" cancer cells are then randomly positioned on the lattice.

Preliminary statistical analyses (Chi-Square and Anderson-Darling test) on the nodule diameters coming from the left lung of 8 mice under the null-hypothesis of normality or log-normality [15] showed that neither of the two distributions approaches *in vivo *data.

Observing *in vivo *results and taking into account the "one cell-one nodule" hypothesis, we deduced that distinct groups of cells originating from the same progenitor represent different populations, each one with its own growth rate. Many factors, and in particular a non-homogeneous distribution of nutrients in lungs, can determine these different growth rates for the nodules.

The reaction-diffusion model for the growth of tumors by Ferreira et. al. showed that using nutrients [16] the growth in time of the cancer approached to a Gompertzian growth. Results by Kendall [15] showed indeed that Gompertzian growth hypothesis fits well with experimental data coming from distributions of human metastases.

Since the distribution of nutrients in the lungs is neither homogeneous nor known, we therefore supposed that nutrients would lead to Gompertzian growths in time and hence we reproduced the growth kinetics of the nodules using the Gompertz growth law [17].

The Gompertz law, introduced in 1825 by Benjamin Gompertz, is a sigmoid function suitable for describing populations growths. The law uses two factors: a growth factor that decreases in time and a constant mortality factor. Thanks to the growth factor deceleration, the dimension of the population tends asymptotically to a certain threshold (the *carrying capacity*). This model is particularly suitable for the following phenomena:

• Mobile phone uptake, where costs were initially high (so uptake was slow), followed by a period of rapid growth, followed by a slowing of uptake as saturation was reached.

• Population in a confined space, as birth rates first increase and then slow as resource limits are reached.

• Modeling of the growth of tumors, where the approaching of the asymptote usually represents the presence of anti-angiogenic growth factors.

Let *x*(*t*) be the number of cancer cells at time *t*, the differential form of the law is given by:

$$\frac{dx(t)}{dt}=ax(t)-bx(t)\cdot \ln\left(x(t)\right)$$

where *a *represents the intrinsic grow factor of the tumor, usually connected to the nutrient availability, and *b *is the mortality factor. Having as an initial condition *x*(0) = *x*_0_, the solution of the equation is:

$$x(t)=e^{\frac{a}{b}}e^{-\left(\frac{a}{b}-\ln x_{0}\right)e^{-bt}}=x_{0}^{e^{-bt}}e^{\frac{a}{b}}(1-e^{-bt})$$

Other formulations use the following form:

$$x(t)=x_{0}e^{\frac{a}{b}}(1-e^{-bt})$$

As previously stated, biologists supposed for the problem we are dealing with that every metastatic nodule originates from a single "progenitor" cancer cell (*x*_0 _= 1). For this reason the coefficient *x*_0 _can be removed. The model can therefore be simplified to:

$$x(t)=e^{\frac{a}{b}}(1-e^{-bt})$$

To model *in silico *the same nodule sizes distributions of the *in vivo *experiment we then decided to use the inverse transform sampling method [18]. Starting from a random variable *u *uniformly distributed in 0[1], the method allows us to obtain a random variable *X *distributed according to some desired experimental data.

The algorithm of the method is as follows:

• Build the cumulative distribution function *F*(*x*) using experimental data;

• Consider ϕ(*y*) = *F*^-1^(*x*);

• Generate a random value *u *uniformly distributed in the range [0, 1];

• Return *r *= ϕ(*u*) distributed according to experimental data.

Supposing that we want to simulate *k *metastatic nodules, we can now generate *k *random nodule measures distributed according to experimental data. nodules have a spherical form, we can estimate the number of cells *x *contained in a nodule with a diameter *r *as follows:

$$x=\frac{{\left(\frac{r}{2}\right)}^{3}}{{\left(\frac{d}{2}\right)}^{3}}$$

where *d *represents the mean diameter of a TuBo cancer cell.

Starting from *x*, we have then to find *a *and *b *in a such way that, using the Gompertz law, the diameter of the nodule at the end of the simulation should be near to the expected value.

The knowledge of *x *is not enough for our aim since we have two unknown variables and just one equation, and this leads to an infinite set of possibilities. For this reason Biologists supposed that, at the end of the experiment, the nodules reached only 50% of the maximum diameter they can have before approaching close to their carrying capacity. Applying the logarithm function to both members, the previous equation translates into:

$$\ln x(t)=\frac{a}{b}(1-e^{-bt})$$

Since each time-step is 8 hours and the experiment lasts for 32 days, we need *t*' = 96 time-steps to complete the simulation.

As previously stated, the law also tends asymptotically to its carrying capacity. If we call x* the number of cells of a nodule at its carrying capacity, the following limit holds:

$$\underset{t\to \infty }{\lim}e^{\frac{a}{b}(1-e^{-bt})}=x^{*}$$

Moreover, since the term *e*^-*bt *^→ 0 for *t *→ ∞, we can suppose that for *t *≫ *t*' the following statement holds:

$$\frac{a}{b}\approx \ln x^{*}$$

Proceeding with substitution we have:

$$\frac{\ln x}{\ln x^{*}}=(1-e^{-bt^{\prime }})$$

and finally:

$$\begin{array}{l} b=-\frac{\ln\left(1-\frac{\ln x}{\ln x^{*}}\right)}{t^{\prime }}\\ \;a=\left[\frac{\ln(x)\cdot b}{1-e^{-bt^{\prime }}}\right] \end{array}$$

The obtained *k *parameters (*a*_*k*_, *b*_*k*_) are associated at time-step 0 to the *k *randomly positioned cancer cells. At any time-step the Gompertz law is used to compute the duplication rates that cancer cells belonging to the specific nodule should have.

In particular, starting from the differential form of the law

$$\frac{dx_{j}(t)}{dt}=a_{j}x(t)-b_{j}x_{j}(t)\cdot \ln\left(x(t)\right)$$

we discretize the equation with the forward Euler method obtaining

$$x_{j}^{t+1}-x_{j}^{t}=\Delta t(x_{j}^{t}(a_{j}-b_{j}\cdot \ln(x_{j}^{t}))).$$

Having discrete time-steps, we can suppose Δ*t *= 1. If we divide all the members by $x_{j}^{t}$ we have:

$$w_{j}=\frac{x_{j}^{t+1}-x_{j}^{t}}{x_{j}^{t}}=(a_{j}-b_{j}\cdot \ln(x_{j}^{t}))$$

Where the index *j *identifies the nodule (or the population) *j*, *j *∈ 1 ... *k*, *w_j _*represents the duplication ratio for all the cells belonging to the nodule *j *and $x_{{}^{j}}^{t+1}$and $x_{j}^{t}$ are the number of cancer cells of the population *j *at time-step *t *+ 1 and *t*, respectively.

### Tuning and validation of the model

Prior to use the model as in silico wet lab, it has to be tuned and validated against existing in vivo experiments. All models have a certain number of parameters which can be freely chosen in a certain range. Biological knowledge has been used to guess reasonable initial ranges. Then fine tuning has been done in such a way that in silico experiments fit in vivo ones. For the reproduction of the metastatic growth pattern it has been possible to utilize experimental data on the distribution of nodules in sizes (diameters) coming from untreated mice. Thus for all scenarios the number of nodules at the end of the "in vivo" experiment has been used.

MetastaSim describes the metastatic growth pattern and immune response elicited by the vaccine against it for a single mouse. Parameters tuning must entitle as result that the simulator, applied to mice with different vaccine protocols, gives as result a reliable representation of the "in vivo" experiment. Note that immune system behavior should agree with biological knowledge. For this reason major entities mean plots were also submitted to biologists and checked for their approval.

The tuning procedure was done using few, randomly selected, individual mice. Parameters are varied under a certain range and then simulations are executed on the sample set. Obtained results are then checked. When a reasonable tuning has been found, "in silico" validation of the model has been done using the following experimental procedure:

• Generate a large population of individual mice, each one with a different random seed which will determine different probabilistic chain events.

• Randomly extract from the population two statistical samples of 100 individual mice to perform numerical experiments.

• Simulate all the scenarios on the two sample sets.

Note here that none of the mice used during the tuning procedure has been used also for validation.

### The treated scenarios

The tests have been executed on two different mice samples, each one composed by 100 virtual mice. For every mice set the untreated mice as well as the two treated mice scenarios (vaccination started at day 1 with Triplex+1 protocol and at day 7 with Triplex+7 protocol respectively) have been reproduced. Since MetastaSim simulates the frontal ventral surface of the left lung of mice, the initial number of metastases already settled in the lungs at the beginning of the simulation has been estimated using experimental data and has been set to 35. The number metastases has been taken as the outcome of the simulations for all the scenarios.

Median values relative to the two treated mice scenarios have been then compared against those coming from the untreated mice scenario and the percentages of prevented metastases (in respect to the untreated scenario) have been estimated, thus following the same procedure used "in vivo" [8].

### Evaluation of "in-silico" predicted protocols

The MetastaSim simulator usually requires no more than 5 minutes to test a protocol on a single mouse. An upper bound for the required total CPU time is ≈ 100·512·5 ≈ 177.8 days on a single cpu computer. It is possible to overcome this time limit by running multiple simulations at the same time using an HPC infrastructure. Resources from the CINECA Italian supercomputing center (BCX cluster) have been then used for the purpose, launching 8 different MPI jobs (each one requiring 128 cores) for approximately 4 hours. The total employed time has been ≈ 4000 hours, which corresponds to 167.7 days on a single cpu computer.

In order to establish whether a protocol is better than another, an order criterion (a fitness or rank function) must be used. For this reason the following rank function has been used:

Let N* ⊂ **R **be the set which contains all the possible values *N_i _*described before, and let **T **⊂ **R **be the set of all possible number of injections *T_i _*for each protocol *i*. Each protocol *i *can be described as a point (*N_i_*, *T_i_*) in the space (N*, **T**). Ideally the optimum protocol lies in the origin (0, 0), since it is able to entitle 0 metastatic nodules with 0 injections. Of course such a protocol does not exist. However the best protocols will be the ones whose distance from the origin is minimum. The following sort criterion is then used:

$$r_{i}=\sqrt{N_{i}^{2}+{\left(k_{3}\cdot T_{i}\right)}^{2}}$$

where *k*_3 _is a fixed constant used to properly scale **T**. The goal of this exhaustive search is to find protocols with the same protection rate as "Triplex+1" protocol but with less injections, so protocol prevention rates must be favored in respect to the number of injections. For this reason *k*_3 _has been empirically set to 15.

## Results and Discussion

### Results

#### Validation of the untreated mice scenario

For the untreated mice scenario, results coming from *in silico *and *in vivo *tests were compared using the Kolmogorov-Smirnov test in its two-sample variant [19].

We tested the mean *in vivo *nodule sizes distribution (coming from 8 different mice) against 8 different *in silico *distributions. Nodule measurements of the *in silico *distributions were estimated starting from the number of cancer cells of each nodule and supposing spherical form for both cells and nodules.

**Figure 2:**
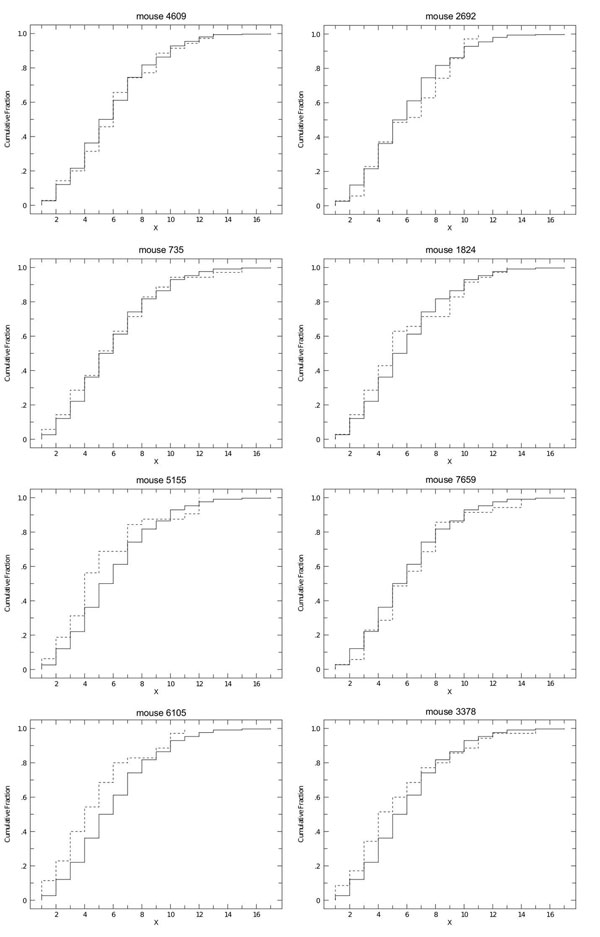
**Cumulative fraction plots for the in vivo and in silico untreated mice distributions**. Comparison of the cumulative fraction plots for the in vivo and the in silico distributions for the untreated mouse case. The "X" axis represents the (estimated for the in silico experiments) diameter of metastatic nodules expressed in ocular micrometer notches. The solid line represents the cumulative fraction plot for the in vivo experiment; the dashed line represents the cumulative fraction plot obtained for the in silico experiments.

**Figure 3:**
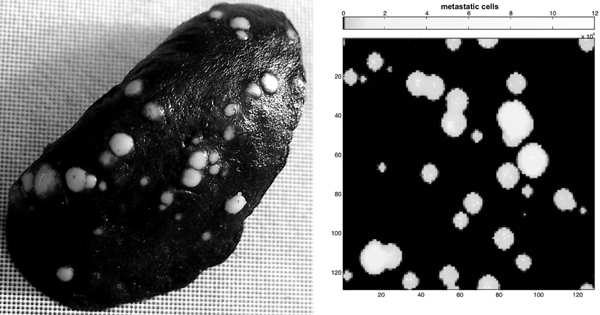
**Nodules distributions in in vivo and in silico experiments**. Examples of nodules distributions obtained *in vivo *(l.s.) and *in silico *(r.s.).

Using a standard significance level *α *= 0.05, for none of the tests we were able to reject the null hypothesis that the two samples are drawn from the same distribution (see table 1, P column). Moreover the maximum distances between the two distributions (table 1, D column) are usually very limited, suggesting that the *in silico *obtained nodule size distributions are in good agreement with the *in vivo *ones. This result can also be observed in figure 2, where we compare the cumulative fraction plot of the in vivo experiment against the ones obtained in the in silico experiments. In figure 3 we show an example of the nodules spatial distributions obtained *in vivo *and *in silico*.

**Table 1:** Results from the two-sample Kolmogorov-Smirnov statistical test

MOUSE	Reject	P	D
4609	No	1	0.0485
2692	No	0.491	0.1451
735	No	0.997	0.0700
1824	No	0.877	0.1027
5155	No	0.113	0.2087
7659	No	0.990	0.0770
6105	No	0.212	0.1843
3378	No	0.435	0.1515

The first column represents the random seed of a given mouse. The second column reports the result of the test (whether the null hypothesis is rejected), the third column (P) shows the value returned by the test (to be compared to the significance level), and the last column (D) the maximum distance between the two distributions (maximum norm).

#### Validation of the treated scenarios

The Kolmogorov-Smirnov test was not used for testing the nodules distributions coming from simulations of "Triplex+1" and "Triplex+7" vaccine protocols. The biggest problem here is represented by the small number of nodules arising in vaccinated mice. In order to execute any statistical test, a sample set should be big enough to be considered significative, i.e., as a valid representation of the phenomenon. Speaking of nodules distributions when the "in vivo" scenario has a too limited number of nodules, if none (as in the case of mice treated with "Triplex+1" protocol), makes nonsense.

To validate results coming from the "in silico" experiment against the "in vivo" results presented in [8], MetastaSim has been executed on two different mice sample sets simulating all the three scenarios: untreated mice, mice treated with the Triplex+1 protocol and mice treated with the Triplex+7 protocol. Then the number of arising nodules has been taken as the outcome. The median value for each experiment was considered and the percentage of prevented nodules has been estimated and compared with the "in silico" results. The methodology used here is similar to those used by biologists in their experiment.

For each scenario the number visible nodules for each mouse has been taken as the outcome of the experiment, then the median has been taken into account. In the "in vivo" experiment, the untreated mice sample set presented a median of visible nodules > 200, whereas the mice sample set treated with a twice a week vaccination cycle started at day 1 presented a median value of 3 visible nodules, showing ≈ 99% reduction of the metastatic burden [8]. In the "in silico" experiment, the untreated scenario presented a median number of visible metastases of 30 and 29 for set I and set II respectively. An "in silico" 99% reduction of nodules would mean that only ≈ 1 mouse out of three would have evidences of the metastatic development. This would also entitle a median value of 0. On the other hand such a value could be easily mistaken to complete prevention of metastases (i.e., 100% prevention). To provide a more accurate estimation in the number of prevented metastases, the mean number of metastases has been taken also into account for this scenario.

Mice whose vaccination started at day 1 showed a median of 0 for both the sample sets. Mean values for sample set I and sample set II are 0.33 and 0.21 respectively, denoting a perfect agreement between "in silico" and "in vivo" prevented metastases percentages.

Mice whose vaccination started at day 7 showed a median of 5 for both the sample sets. Comparison with medians in untreated mice (30 and 29 for set II and set II respectively) suggests a percentage range of 82,7% - 83,3% in the number of prevented metastases, against an "in vivo" obtained percentage (≥ 87%). This indicates a good agreement between "in silico" and "in vivo" prevented metastases percentages even for the Triplex+7 protocol. Results are shown in table 2.

**Table 2:** Results from validation of the treated scenarios

Vaccination	Injected Cells	Median	Range	Estimated Prevention
**"In vivo" experiment**				

No	-	> 200	134 to > 200	-
Day 1	-	3	0 - 27	≈ 99%
Day 7	-	26	1 - 165	≥ 87%

**"In silico" 100 mice Sample I**				

No	35	30	23 - 34	-
Day 1	35	0 (mean 0.33)	0 - 2	≈ 99%
Day 7	35	5	0 - 12	≥ 83,3%

**"In silico" 100 mice Sample II**				

No	35	29	23 - 34	-
Day 1	35	0 (mean 0.21)	0 - 2	> 99%
Day 7	35	5	1 - 11	≈ 82,7%

Results obtained in the "in vivo" experiment and in the "in silico" experiments on mice Set I and II using the following vaccine protocols: "Untreated" (No vaccination), "Triplex+1" (Day 1) and "Triplex+7" (Day 7). The "Injected" column represents the number of cancer cells positioned in the lattice in the "In silico" experiments at the beginning of the simulation. The "Median" column shows the nodule medians obtained at the end of the experiments. The "Estimated prevention" column indicates the efficacy of a protocol in vaccinated mice in respect to untreated mice, and it is computed comparing the medians obtained in vaccinated mice against the medians obtained in untreated mice.

#### Exhaustive search for optimal vaccine schedules

The search space for the problem counts 2^9 ^= 512 possible different protocols and it is therefore very limited. In this case it is advisable to use an exhaustive search rather than an optimization technique, as shown in [20,21] for the SimTriplex model, to find an optimal protocol.

However it is important to note here that the exhaustive search should not be executed on a single mouse. We have indeed chosen to execute the exhaustive search on 100 different mice. Results relative to the first 20 best protocols are shown in table 3.

**Table 3:** Classification of best protocols found using the exhaustive search

Protocol ID	Protocol	Injections	Median
87	001010111	5	0
103	001100111	5	0

119	001110111	6	0
183	010110111	6	0
215	011010111	6	0
311	100110111	6	0
343	101010111	6	0

375	101110111	7	0
471	111010111	7	0

255	011111111	8	0
447	110111111	8	0
503	111110111	8	0

511	111111111	9	0
47	000101111	5	0,5
199	011000111	5	0,5

231	011100111	6	0,5
303	100101111	6	0,5

127	001111111	7	0,5

7	000000111	3	1
15	000001111	4	1

The "Protocol ID" column identifies the protocol used. The "Protocol" column represents the protocol in binary format (i.e., 1/0 equals to administration/no administration at the desired day). Rightmost bits represent earlier vaccinations (at the beginning of the experiment); leftmost bits represent later vaccinations (at the end of the experiment). The "Injections" column counts the number of injections of the desired protocol. The "Median" column shows the obtained median relative to the total number of nodules, and it is determined over a set of 100 virtual mice.

As the outcome of the experiment the total number of metastases $n_{i}^{j}$ has for every protocol *i *= 1 ... 512 and every mouse *j *= 1 ... 100 has been taken into account. The median numbers *N_i _*of the number of formed metastases has been then calculated for every protocol *i *on $n_{i}^{j}$.

A first analysis of the results confirmed biologists expectations: protocols counting more early vaccine administrations give better results than protocols with late vaccinations. For example, using 4 administrations, protocol 15 = 111100000 is able to elicit almost complete protection with *N_i_*, whereas protocol 480 = 000001111 turned out to be almost useless with *N_i _*= 24.

**Figure 4:**
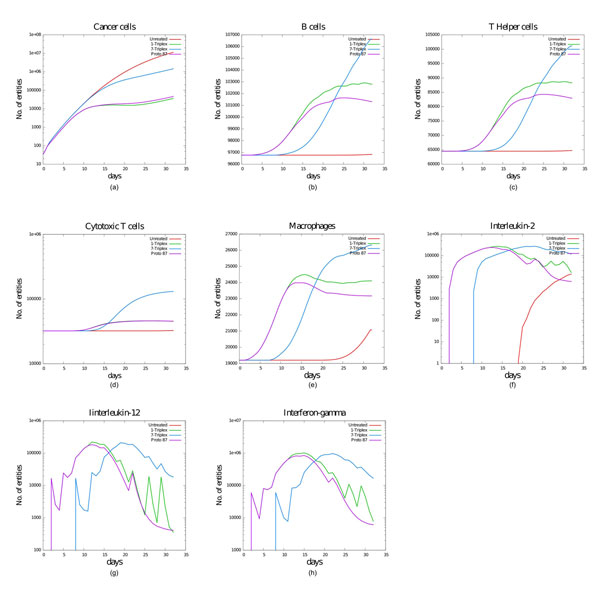
**Comparison of behaviors using 1+Triplex and Protocol 87**. Mean dynamics of relevant entities in untreated (red line) and treated mice using Triplex+1 (blue line), Triplex+7 (green line) and protocol 87 (purple line). From left to right, up to down: Cancer cells (CC) (a), B cells (B) (b), TH cells (TH) (c), TC cells (TC) (d), macrophages (MP) (e), interleukin-2 (IL-2) (f), interleukin-12 (IL-12) (g) and interferon-γ (INF-g) (h). CC, Ag, IL-2, IL-12 and INF-g plots are presented on a logarithmic scale to improve comparison.

Results also show that it is possible, using protocols 87 and 103, to achieve "in silico" (with just 5 injections) the same protection elicited by the 9-injections protocol "Triplex+1". Figure 4 summarizes the mean behaviors (computed on 100 mice) for the most important entities in mice without treatment, using protocols "Triplex+1", "Triplex+7" and protocol 87 which consists of 5 injections. From figure 4 it is possible to see that the 5-injections protocol 87 is able to entitle similar immune response as the "Triplex+1" protocol. B lymphocytes (B) (b), cytotoxic T cells (TC) (d), T helper cells (TH) (c), and Macrophages (MP) (e) plots show how the vaccine is able to favor immune system responses thanks to the earlier appearance of the antigen (Ag) and of interleukin 12 (IL-12) (g). Also interleukin 2 (IL-2) (f) appears earlier and in greater quantities. The lack of the initial two injections shows how the "chemotaxis-driven" stronger immune response entitled with the use of protocol "Triplex+7" (see in particular the cytotoxic T cells dynamics, boosted also by the "TC activation" interaction with a bigger number of Cancer Cells) is however unable to effectively deal with the exponential growth of the metastatic nodules.

## Discussion

We have presented MetastaSim, a computational model to be used as in silico wet-lab. The in silico validation showed a good agreement with the in vivo results [8] (tables 1 and 2), demonstrating that the model is able to coherently reproduce the in vivo experiments. The model has been then used to try to predict protocols capable of assuring the same protection entitled with the Triplex+1 protocol but with fewer injections. An exhaustive search for any optimal protocol has been performed. Results showed that it is possible to obtain in silico a reduction of approximatively 45% in the number of vaccinations (table 3). An important thought rises up from analyzing the results presented in table 3. Most of the protocols presented there share a similar vaccination strategy which is composed by a boost of three vaccine injections, a period of rest, and then a series of vaccine recalls that are somewhat equally spaced. The model suggests that any optimal protocol for preventing lung metastases formation should be therefore composed by an initial massive vaccine dosage followed by few vaccine recalls. Even if this is a well-known vaccination strategy in immunology, since it is commonly used for many infectious diseases such as tetanus and hepatitis-B, it can be still considered a relevant result in the field of cancer-vaccines immunotherapy. Another fact that should be noted here is that protocol 7, which counts only the initial boost of three injections, is able to entitle a high level of protection against metastases. This underlines the importance of the first boost of vaccinations, and partially justifies the not so optimal prevention rates given by protocol "Triplex+7", which does not include the initial boost. To deal with an exponential-like growth of cancer cells (like a Gompertz growth [17]), a significant vaccination should be a administered in time, in order to train the immune system to engage a fight against cancer cells and to kill them before their number grows enormously. Later vaccine recalls can indeed be useful to keep immunity at a high level in order to readily react against escaped or dormant cancer cells.

## Conclusions

The metastatic process is extremely complex and inefficient. However, in spite of their inefficiency, metastases are a major cause of mortality in cancer patients.

Innovative approaches, both on the therapeutic and immunopreventive side, are nowadays the major hopes for eradicating this appalling disease. Mathematical and computational models to simulate and to better understand "in vivo" experiments can represent a valuable help to biologists and physicians. Nevertheless immune system complexity requires non-conventional simulation techniques to correctly model and simulate the immune behavior. The MetastaSim computational model represents one of these techniques.

Using an agent-based approach, we model the immune system at the cellular-level without excluding important features and phenomena observed at the molecular scale, like receptors, that are vital to mimic important biological processes such as clonal selection. Starting from low-level components and interactions, the model is then able to show population behaviors at a higher level.

The Gompertz law (in its differential form) is also used to reproduce the growth patten in time of metastatic nodules, making the model an ABM-ODE hybrid model.

The model has been tuned using "in vivo" experimental data, and the results confirm that there is a good agreement between the "in silico" and "in vivo" experiment. It has been then used as an "in silico" wet-lab to find an optimal protocol, capable of assuring almost complete prevention of lung metastases formation with fewer injections. Results showed that it is possible, with a reduction of approximatively 45% in the number of vaccine injections, to achieve the same protection entitled by the "Triplex+1" chronic protocol. This may represent a remarkable result since from a point of view of translational medicine towards clinical Phase-I trials in humans, it is usually desirable to minimize the number of vaccinations, to minimize the risk of undesirable effects and to speed-up the entire process.

Moreover the model suggested an optimal vaccination scheme for preventing lung metastases formation. According to the obtained results, the vaccine strategy should be composed by an early significant vaccine dosage followed by few vaccine recalls in order to deal with cancer cells' exponential growth. Such a strategy can be then tested and adopted instead a chronic "linear" strategy (i.e., the same equally spaced vaccination cycles for the entire experiment), since it could allow similar optimal results with a reduction in the number of vaccinations.

These results can be then used and verified in future "in vivo" experiments, and their outcome can be used to further improve and refine the model.

## Authors' contributions

MP developed the MetastaSim model.

FP provided the general framework of agent based modeling and supervised the test phase of MetastaSim model.

A.P. and G.N. performed the in vivo therapeutic experiments;

P.N. supervised in vivo experiments and performed metastasis counts;

P-L.L. acted as a liaison between cancer researchers and informatics and collaborate to the design of the mathematical model.

S.M. conceived the application of an immunological simulator to cancer vaccines. He provided mathematical knowledge.

All authors read and approved the final manuscript.
